# Assessment of Cell Disruption Methods in an Integrated Multi-Product Biorefinery for *Nannochloropsis oceanica*: From Process Design to Economic Analysis

**DOI:** 10.3390/md24070245

**Published:** 2026-07-14

**Authors:** Pedro Cunha, Bernardo Carvalho, Mariam Kholany, Hugo Pereira, João Varela

**Affiliations:** 1 GreenCoLab—Associação Oceano Verde, Universidade do Algarve, Campus de Gambelas, 8005-139 Faro, Portugal; pedrocunha@greencolab.com (P.C.); bernardocarvalho@greencolab.com (B.C.); mariamkholany@greencolab.com (M.K.); hugopereira@greencolab.com (H.P.); 2 Allmicroalgae-Natural Products, Rua 25 de Abril, 2445-413 Alcobaca, Portugal; 3 Center of Marine Sciences, Universidade do Algarve, Campus de Gambelas, 8005-139 Faro, Portugal

**Keywords:** microalgae, biorefinery, multi-product, process integration, membrane filtration, ethanolic extraction, cost-effectiveness

## Abstract

Microalgae are bioresources with significant potential within a sustainable, circular, bio-economy. However, high production costs have limited the widespread use of algae biomass. This study aimed to develop a multi-product biorefinery for *Nannochloropsis oceanica* that generates multiple revenue streams from the biomass, thereby enhancing the economic viability of algal production. The effectiveness of cell wall disruption using high-pressure homogenization and enzymatic hydrolysis was evaluated. Enzymatic hydrolysis solubilized nearly half (48.2 ± 1.5%) of the dry cell weight, compared to only 27.3 ± 3.2% with high-pressure homogenization, resulting in more concentrated water-soluble fractions and significantly higher protein extraction yields. Lipid extracts obtained after enzymatic hydrolysis had higher lipid (72.0 ± 5.3% *w*/*w*) and eicosapentaenoic acid (28.1 ± 6.9% *w*/*w*) contents than those from high-pressure homogenization (38.8 ± 6.1% *w*/*w* lipids; 9.1 ± 0.6% *w*/*w* eicosapentaenoic acid), despite similar lipid extraction yields (around 30%). Increasing the ethanol volumetric ratio from 58% to 75% *v*/*v* significantly improved lipid extraction yields (57.4 ± 3.1%) in the enzymatic hydrolysis-based biorefinery, with even higher yields observed upon scaling up (70.1%). All fractions, including lipid extracts, exhibited a balanced essential amino acid profile that exceeded the WHO/FAO/UNU-recommended values. A preliminary economic analysis indicated that lipid production was more cost-effective when cells were permeabilized by enzymatic hydrolysis than by high-pressure homogenization.

## 1. Introduction

Biorefineries have been gaining momentum as the need to transition from a fossil-based to a more sustainable, bio-based economy becomes apparent. Instead of crude petroleum, biorefineries use biomass as feedstock and fractionate it into its different components [1]. The purpose of multi-product biorefineries is to produce multiple products from a single biomass, thereby extracting the maximum value from the initial raw material [1]. Despite being a hot topic nowadays, biorefineries have long existed in industries such as corn, dairy, and lignocellulosic. However, for microalgal biomass, multi-product biorefineries are a relatively recent concept [2]. So far, downstream processing has focused on whole-cell production or on the extraction and purification of the most valuable biomass component, treating the remaining parts as a residue [3]. This approach is valid for specialty products with very high market value and reflects the fact that microalgae commercialization is mainly restricted to niche markets [4]. The high capital and operational costs associated with microalgae cultivation and processing have hindered the microalgae industry from reaching its full potential [5]. Creating multiple revenue streams from a single microalgal biomass through multi-product biorefineries is crucial for closing the gap between market value and production costs, thereby contributing to the economic viability of commercializing lower-value products [4]. Integrating all the biorefinery unit processes, along with the upstream and establishing trade-offs—such as purity vs. yield and cost of production vs. product value—from the beginning of process design until the optimization stage is fundamental for the cost-efficiency of the biorefinery [1,4,6]. Additionally, mild processing techniques that preserve the original properties (structure and functionality) of biomass components must be considered for optimal multi-product valorization [1,3].

The wide biochemical diversity of microalgae, along with their unique characteristics, has garnered the attention of the scientific community and industry. These characteristics include their role as primary producers, their ability to grow on non-arable land, their high growth rates, and their lower land and water footprints than those of higher plants [3]. The genus *Nannochloropsis* has been a subject of particular interest because it comprises highly robust species well adapted for year-round, large-scale outdoor cultivation and high mass productivity [7]. The high lipogenic potential of these species has made them a focus of research on biofuel production [8]. However, their rich biochemical profile, comprising high-value molecules, such as omega-3 polyunsaturated fatty acids (PUFAs), mainly eicosapentaenoic acid (EPA), antioxidants (carotenoids and phenolic compounds), pigments, and water-soluble polysaccharides, in addition to their high protein content, offers many more biorefining possibilities beyond energy production [9,10,11].

*Nannochloropsis* cells are highly resistant to mechanical stress due to their small size (typically 2 to 8 μm [12]) and their thick, multilayered cell wall [13,14]. The inner cell wall comprises a network of tightly packed ordered arrays of cellulose microfibrils cross-linked by hemicellulose molecules, and is surrounded by highly resistant aliphatic algaenan polymers, which constitute the outer cell wall [14,15,16]. These characteristics contribute to the cell wall’s rigidity, which significantly hinders the disruption of *Nannochloropsis* cells, often the first step in algal biorefineries, and access to their intracellular metabolites.

High-pressure homogenization (HPH), along with bead milling, is the most commonly used method for disrupting microalgae at an industrial scale [17]. However, it has difficulty breaking rigid cell walls, generates fine cell debris, and raises concerns about its mildness [17]. Unlike HPH, enzymatic hydrolysis (EH) is a non-mechanical method distinguished by its biological specificity (targeting specific bonds in the cell wall) and mild operating conditions, making it a promising alternative for permeabilizing rigid cell walls [17].

**Figure 1:**
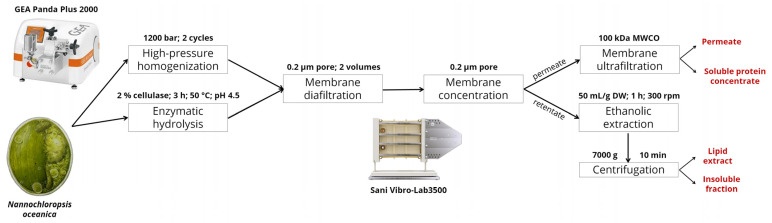
Diagram of the two biorefinery routes tested. High-pressure homogenization and enzymatic hydrolysis were used for cell disruption and integrated with a multi-product fractionation process that employed both membrane filtration and ethanolic extraction. Four final fractions resulted from the cascade processing: (1) a soluble concentrated fraction, (2) the remaining soluble components (permeate), (3) a lipid extract, and (4) an insoluble fraction.

This work focused on assessing the effectiveness of these two techniques in disrupting *Nannochloropsis oceanica* cells within an integrated multi-product biorefinery. While most research focuses solely on comparing different disruption methods and quantifying the percentage of disrupted cells, this work evaluated disruption as part of an integrated processing pipeline to valorize *N. oceanica* biomass. Upon cell disruption, membrane filtration and ethanolic extraction were used to fractionate the lysed biomass, yielding a protein-rich water-soluble concentrate and a purified lipid extract (Figure 1). Detailed biochemical characterization of the produced fractions and mass-balance calculations were performed. Furthermore, an economic analysis of various biorefinery scenarios enabled us to select the most cost-effective cascade of operations and determine the optimal operating conditions.

## 2. Results

This work aimed to develop an integrated downstream processing pipeline capable of fractionating *N. oceanica* biomass into multiple ingredients to maximize its value. Given the high mechanical resistance of *Nannochloropsis*’s cell wall, efficient cell disruption is crucial before fractionation. Therefore, enzymatic hydrolysis with cellulase, specifically targeting the cellulose-rich *Nannochloropsis* cell wall, was compared with high-pressure homogenization within an integrated biorefinery. The fractionation process was designed to produce a concentrated, protein-rich, soluble fraction and a lipid extract with high purity and high EPA content.

### 2.1. Optimization of Protein Quantification

Given the complexity of accurate protein quantification, an optimization was performed before the mass balance calculation. This was done by analyzing only the samples from one of the three replicates of the biorefinery processes because of the costly and time-consuming nature of amino acid profiling.

The protein content of the water-soluble fractions was evaluated using two different methods: the modified Lowry method and total amino acid determination (Table 1). The latter was used as the benchmark to assess the reliability of the other methods, as it is the most rigorous analytical method for protein quantification. The modified Lowry method tended to underestimate the results compared to the total amino acid content determination method. This underestimation was particularly pronounced when applying HPH cell disruption. Using EH, the results were much closer for both methods. The protein content of UF retentate (for EH-based biorefinery) seemed to be higher using the Lowry method than the total amino acid quantification. This alternate trend in the results suggests that the Lowry method is inaccurate for protein quantification.

**Figure 2:**
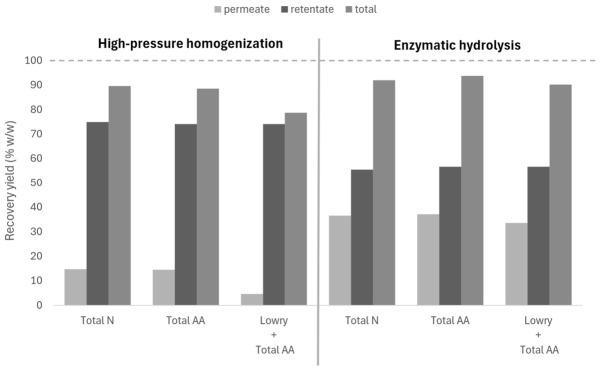
Test assay performed to optimize the protein quantification in each fraction. The protein mass balance to microfiltration in both biorefineries tested is shown (*n* = 1). Three methods to determine the total protein content of each fraction were compared: total nitrogen for all fractions; total amino acids for all fractions; and Lowry for water-soluble fractions and total amino acids for the remaining fractions. The protein recovery yield in the permeate and retentate fractions was calculated using the protein content of the lysates as the reference. The sum of the permeate and retentate yields is lower than 100% due to protein adsorption on membranes (fouling) during microfiltration [18].

Based on the protein content results, mass balances for microfiltration were calculated with different approaches (Figure 2), namely: (1) using the total nitrogen content of each fraction to estimate the protein distribution; (2) evaluating the protein content of each fraction as the sum of all amino acid residues; and (3) determining the protein content of the soluble fractions (MF permeates) using the Lowry method, while quantifying the remaining fractions (homogenate, hydrolysate, and MF retentates) through total amino acid quantification. The third approach underestimated protein content, particularly in the HPH-based biorefinery (approximately 10% less total protein was quantified compared with methods 1 and 2). This discrepancy is attributed to the lower efficiency of the Lowry method for quantifying protein in soluble fractions, as previously mentioned. In contrast, the mass balances obtained from total nitrogen quantification were very similar to those derived from total amino acid quantification for both biorefineries (HPH and EH). Thus, nitrogen-to-protein conversion factors were calculated from total nitrogen and total amino acid content (Table 2). The nitrogen-to-protein ratio was very similar across most fractions, except for the UF retentate of the EH biorefinery, which showed a significantly lower value. Excluding this retentate (nitrogen-to-protein ratio of 3.92), an average value of 5.35 ± 0.1 was calculated from the remaining fractions. This average nitrogen-to-protein ratio and the nitrogen content of each fraction were then used to estimate the protein content of every fraction produced in all three replicates of both biorefineries tested.

### 2.2. Cell Disruption Efficiency

**Figure 3:**
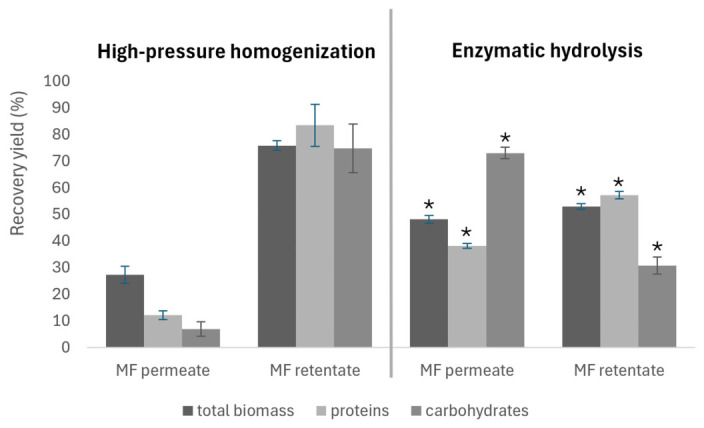
Total biomass, protein, and carbohydrate mass balances of the microfiltration process performed after cell disruption by high-pressure homogenization or hydrolysis to clarify the homogenate/hydrolysate. The recovery yield is calculated relative to the total biomass, protein, and carbohydrates in the lysates. The values are presented as the average and standard deviation of three biological replicates. * Indicates statistically significant differences (*p* < 0.05) between both permeabilization strategies within a fraction.

The efficiency of high-pressure homogenization or enzymatic hydrolysis in disrupting the cell wall of *N. oceanica* was compared by measuring the amount of intracellular components released from cells. To do so, both the permeate and retentate from microfiltration were biochemically characterized, and mass balances for total solids, proteins, and carbohydrates were calculated (Figure 3).

EH was found to be more efficient than HPH for extracting intracellular compounds, with almost half of the total hydrolysate mass (48.2 ± 1.5%) recovered in the MF permeate. In comparison, the permeate mass yield after HPH was only 27.3 ± 3.2%. Similarly, the protein permeate yield obtained after EH (38.0 ± 1.1%) was 3-fold higher than that achieved after HPH (12.3 ± 1.7%). The difference in permeate yields between the two processes was even more pronounced for carbohydrates, with EH reaching 73.0 ± 2.2%, approximately ten times higher than that achieved with HPH (7.0 ± 2.7%). This result was expected, since cellulase was used in the EH process.

### 2.3. Fractionation

After cell permeabilization and lysate clarification, the permeate and retentate obtained by microfiltration were further separated into four fractions (Table 3 and Table 4). The water-soluble fractions (permeate and retentate upon ultrafiltration) obtained after enzymatic hydrolysis were significantly more concentrated than those obtained after high-pressure homogenization, particularly in proteins and carbohydrates. Regarding the EH biorefinery, ultrafiltration produced higher permeate yields (75.0 ± 8.9% for proteins and 74.5 ± 1.5% for carbohydrates) than retentate yields (27.7 ± 7.0% for proteins and 18.9 ± 4.1% for carbohydrates).

The lipid extract obtained after EH was about twice as concentrated as that after HPH. However, lipid extraction yields were similar between the two biorefineries (29.6 ± 4.1% for HPH and 31.2 ± 2.3% for EH). Overall, only 16.6 ± 5.1% and 24.9 ± 5.4% of the initial lipids were recovered in the ethanolic extracts for HPH and EH, respectively. This lipid extraction from the MF retentates resulted in protein enrichment in the remaining insoluble fraction (from 36.9 ± 0.6% to 41.6 ± 1.1% for HPH, and from 34.5 ± 1.3% to 40.0 ± 1.2% for EH). Notably, the insoluble fraction of the HPH biorefinery accumulated significantly more proteins out of the total proteins in the biomass (82.2 ± 6.6%) than that of the EH biorefinery (55.8 ± 3.1%).

### 2.4. Optimization of Lipid Extraction

**Figure 4:**
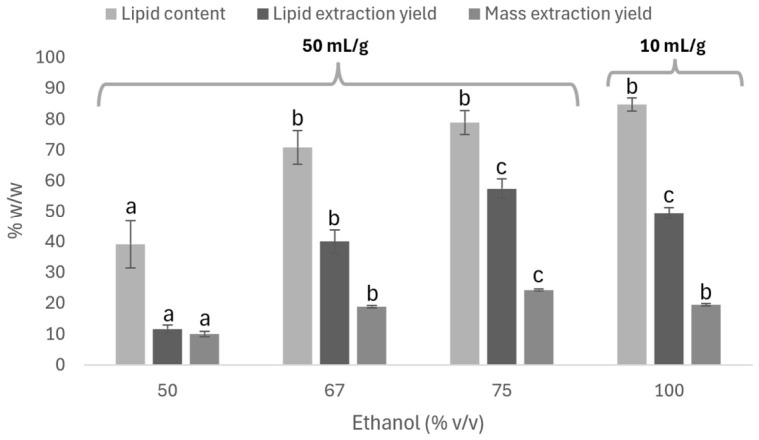
Performance of lipid extraction from the microfiltration retentate (EH biorefinery) in terms of yields and purity of the extracts at different ethanol volumetric concentrations. The values are presented as the average and standard deviation of three replicates. Different letters indicate statistically significant differences (*p* < 0.05) between the different extractions.

Given the low extraction yields, lipid extraction was optimized by varying the ethanol–water volumetric ratio (Figure 4).

Lipid extraction efficiency increased significantly with increasing ethanol volumetric concentration. The yield difference was particularly pronounced when the ethanol–water ratio was increased from 1:1 (50% *v*/*v* ethanol) to 2:1 (67% *v*/*v* ethanol). Extracting lipids from a dry substrate did not further improve extraction efficiency or extract purity. Despite achieving a lipid extraction yield similar to that of the 75% *v*/*v* ethanol extraction, fewer overall compounds were co-extracted, thereby increasing the extraction’s selectivity. Regarding wet lipid extractions, the lower ethanol–water ratio (50% *v*/*v*) extraction was less selective, as lipid and total mass extraction yields were similar. In contrast, for the other two wet lipid extractions, the lipid extraction yield was considerably higher than the total mass extraction yield. This difference explains the lower lipid content observed in the extract obtained with 50% *v*/*v* ethanol.

Based on the results and to avoid an additional drying step before lipid extraction, the 75% *v*/*v* ethanol volumetric concentration was chosen for scaling up. The result was a significantly higher lipid extraction efficiency (70.1%) than under the same conditions at a smaller scale (57.4 ± 3.1%). The purity of the extract and total mass extraction yield were similar for both the larger (80.4% *w*/*w* lipids; 26.4% yield) and smaller scales (78.9 ± 3.9% *w*/*w* lipids; 24.3 ± 0.3% yield).

### 2.5. Biochemical Composition

#### 2.5.1. Fatty Acid Profile

**Figure 5:**
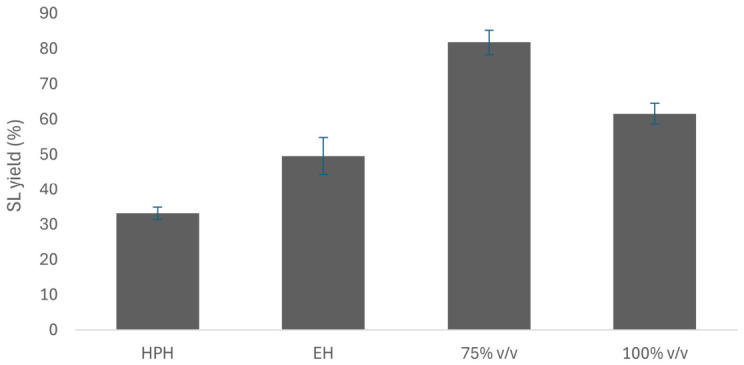
Total saponifiable lipid (SL) yield from ethanolic extractions of both biorefineries (HPH and EH). The SL yields from the 75% *v*/*v* and 100% *v*/*v* ethanol extractions conducted during lipid extraction optimization in the EH biorefinery are also shown. Total SLs in the microfiltration retentate fraction were used as the reference for determining SL extraction yield. The values are presented as the average and standard deviation of two biological replicates.

Total saponifiable lipids (SL) (lipid molecules that comprise fatty acids) and eicosapentaenoic acid (EPA) contents were determined (Table 5). In addition, SL recovery yield from the microfiltration retentate was calculated (Figure 5).

Lipid extracts from the EH biorefinery were significantly richer in SL, particularly EPA, than those derived from the HPH biorefinery. Increasing the ethanol volumetric ratio during lipid extraction in the EH biorefinery did not further enrich the lipid extracts in SL or EPA. However, the SL extraction yield, which was higher for the EH biorefinery (49.6 ± 5.2%) compared with that of the HPH biorefinery (33.2 ± 1.8%), increased even further (81.8 ± 3.5%) when a higher ethanol–water ratio (3 to 1 or 75% *v*/*v* ethanol) was employed. In contrast, extracting lipids directly from dry biomass resulted in a lower SL extraction yield (61.6 ± 3.0%). The polyunsaturated–saturated (PUFA/SFA) fatty acid ratio of the lipid extracts of the EH biorefinery was higher than that of the HPH biorefinery. The most prevalent fatty acids were palmitoleic acid (C16:1), palmitic acid (C16:0), and EPA (C20:5 n-3), accounting for approximately 75% of all fatty acids, followed by myristic acid (C14:0), linoleic acid (C18:2c n-6), oleic acid (C18:1c), and arachidonic acid (C20:4 n-6), which were present in lower amounts. The complete fatty acid profile is shown in Table A1 of Appendix A.

#### 2.5.2. Amino Acid Profile

**Figure 6:**
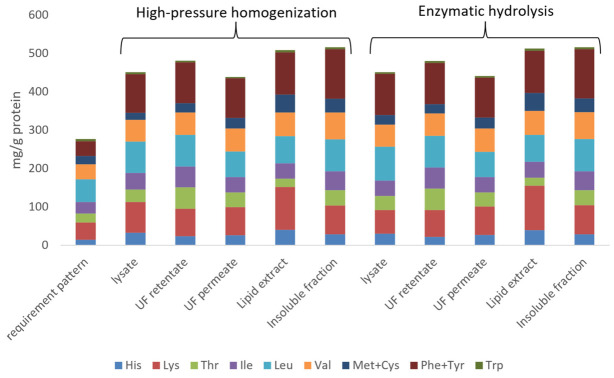
Essential amino acid distribution of the fractions produced in both biorefineries compared to the requirement pattern distribution recommended by WHO/FAO/UNU [19].

The amino acid profiles of selected fractions from both biorefineries were determined (see Table A2 in Appendix A). The content of essential amino acids (EAAs) of each fraction was compared with the requirement pattern values proposed by WHO/FAO/UNU (Figure 6) [19]. The EAAs’ content in every fraction for both biorefineries significantly exceeded the proposed requirement values overall, and for each amino acid except tryptophan. The aromatic amino acids phenylalanine and tyrosine (a metabolic product of phenylalanine catabolism) were the most prevalent in every fraction except the lipid extracts, in which lysine was present at a slightly higher concentration. Tryptophan is the least concentrated EAA in all fractions. Lysate fractionation resulted in fractions with relative EAAs contents similar to the lysates, but with significantly different absolute EAAs contents. Histidine, lysine, and methionine plus cysteine (a metabolic product of methionine catabolism) contents were higher in lipid extracts; threonine and isoleucine contents were higher in UF retentates; and valine and phenylalanine plus tyrosine contents were higher in the insoluble fraction. Overall, the fractions with the highest (insoluble fraction) and lowest (lipid extract) protein contents presented the two largest EEA contents.

### 2.6. Preliminary Economic Analysis

The various biorefinery scenarios, designed to assess different process sequences and processing conditions for producing the lipid extract from *N. oceanica* biomass, were evaluated in terms of specific energy consumption and production costs (Table 6). Scenarios 1 and 2 were compared to evaluate how the extent of dewatering affects the overall energy consumption. Scenarios 2 and 3 were compared to determine which disruption method (HPH or EH) yields a more cost-effective biorefinery. Scenarios 3, 4, and 5 were compared to determine the relevance of reducing water content before lipid extraction on the energy efficiency of downstream processing.

Concentrating the culture harvested from the photobioreactors (assumed to be at 1 g L^−1^) 100-fold (scenario 2) instead of 20-fold (scenario 1) results in an energysaving of 17% during lipid extract production. The energy consumed during dewatering is similar in both cases. Still, the power required for the HPH is approximately five times lower in scenario 2, since the processed volume is also five times smaller.

The specific energy consumption is lower when EH is used (scenario 3) than when HPH is used (scenario 2) for permeabilizing cells, mainly because of the higher lipid extraction yield (40% for EH and 31% for HPH).

Increasing the volumetric ethanol concentration from 67% *v*/*v* (scenario 3) to 75% *v*/*v* (scenario 4) improved the biorefinery’s cost-effectiveness. This was achieved by further concentrating the retentate fraction during microfiltration, increasing the final concentration from 40 g L^−1^ to 60 g L^−1^, while maintaining the ethanol-to-biomass ratio of 50 mL/g-DW. The lower volumetric water concentration increased lipid extraction efficiency from 40% to 57% and reduced the energy required for ethanol recovery via distillation.

Spray-drying the MF retentate, followed by lipid extraction on a dry substrate (scenario 5), did not reduce the production cost of the lipid extract. The energy required for spray-drying and the lower lipid extraction efficiency (49%) compared with scenario 4 offset the energy savings during distillation, because no water was present to vaporize (only the water in the 96% *v*/*v* ethanol azeotropic mixture was present). Overall, scenario 4 was the most cost-effective of all the simulated scenarios. A 10-fold increase in the biorefinery’s production scale relative to scenario 4 reduced production costs by 39%.

## 3. Discussion

### 3.1. Optimization of Protein Quantification

The Lowry spectrophotometric procedure is a colorimetric method based on the reduction of the Folin reagent by aromatic residues and peptide bonds in proteins, producing blue-colored compounds [20]. The presence of other soluble reducing substances that can react with the Folin reagent will interfere with protein quantification, leading to inaccuracies [21]. In addition, choosing a standard protein for calibration similar to the samples’ proteins is critical for the accuracy of the quantification [21]. The reactivity of a standard protein depends on its amino acid composition, as not all amino acids can reduce the Folin reagent equally. The functional groups of amino acids influence the charge and geometry of neighboring atoms, thereby affecting the reactivity of the entire molecule [22]. This becomes particularly challenging in protein fractionation, as the protein profiles of the fractions may change during the process. These two factors were likely responsible for the observed difference in protein contents measured using the Lowry method compared with total amino acid quantification. Spectrophotometric methods can be helpful for relative protein quantification, but are not sufficiently accurate for absolute protein quantification required for mass balance calculations [21].

Accurate protein quantification can be achieved using nitrogen-to-protein conversion factors, as the mass balances based on nitrogen content or total amino acid content were very similar (Figure 2). The nitrogen-to-protein ratios of all fractions from the third replicate of the process (Table 2) were calculated to assess variability among fractions. The values were consistent across most fractions, except for the value corresponding to the UF retentate of the EH biorefinery, which presented a considerably larger percentage of non-protein nitrogen (33.2% of the total nitrogen) compared with all the other fractions. This is likely due to post-translational modifications (including the insertion of nitrogen-containing compounds) on high-molecular-weight structural proteins released during hydrolysis, which were retained by the ultrafiltration membrane [23]. Thus, the average nitrogen-to-protein ratio of 5.35 was used, in combination with the nitrogen content of each fraction, to calculate protein concentration. This is a practical and reliable method for absolute protein quantification, which is much cheaper and less time-consuming than profiling the total amino acid content of each fraction [21].

Although direct amino acid quantification is considered the most accurate method for protein content determination, it does not distinguish between free amino acids and those present in proteins [24]. Consequently, protein overestimation occurs when the percentage of free amino acids from total amino acids is high. Similarly, total nitrogen includes both protein and non-protein nitrogen, which can also lead to protein overestimation when non-protein nitrogen compounds are abundant (using the nitrogen-to-protein conversion factor approach).

### 3.2. Cell Permeabilization Efficiency

*Nannochloropsis* species are characterized by having small cells with thick, cellulosic-rich cell walls. The multilayered, tightly packed cellulose microfibrils, together with the external algaenan layer, render the *Nannochloropsis* cell wall highly resistant to mechanical disruption. Enzymatic hydrolysis with cellulase is a non-mechanical method that directly targets cellulose, the most abundant component in *Nannochloropsis* cell walls. This method proved highly effective, as nearly three-quarters of all carbohydrates were hydrolyzed and recovered in the soluble fraction after clarification. The soluble fraction accounted for almost half of the total biomass and contained 38.0 ± 1.1% of the proteins in the hydrolysate. The hydrolytic action of cellulase was significantly more efficient than high-pressure homogenization at releasing intracellular compounds from *N. oceanica* cells. However, Safi et al. [25] reported lower protein yield with 5% (*w*/*v*) alcalase (35 ± 1%) than with one cycle of HPH at 1500 bar (49 ± 1%) for *Nannochloropsis gaditana*. This demonstrates that selecting the appropriate enzyme based on the composition of the matrix to be hydrolyzed is crucial for hydrolysis efficiency. Safi et al. [26] found that a pressure of 1000 bar was sufficient to release the maximum amount of proteins (around 50% of all proteins in *N. gaditana*). Further increasing the pressure did not improve protein extraction because some proteins are insoluble. In the present work, the protein extraction yield (12.3 ± 1.7%) was significantly lower than previously reported values, indicating that interspecies variability substantially influences cell resistance to disruption.

### 3.3. Fractionation

The purpose of ultrafiltration in these biorefinery processes was to concentrate the soluble fraction obtained from microfiltration, thereby producing a protein concentrate. However, most proteins passed through the membrane into the permeate, and only 26.1 ± 8.9% and 27.7 ± 7.0% of the proteins in the MF permeate were recovered in the retentate at HPH and EH biorefineries, respectively. The hydrolytic action of cellulase resulted in a protein permeate yield of 75.0 ± 8.9% in ultrafiltration, comparable to that obtained by Ribeiro et al. [18] (78.0 ± 0.4%), Safi et al. [25] (71 ± 0%), and Soto-Sierra & Nikolov [27] (73 ± 3%). In all three studies, *Nannochloropsis* sp. or *Nannochloropsis gaditana* biomasses were disrupted mechanically or enzymatically (with alcalase, a highly efficient proteolytic enzyme), and the resulting cell lysates were ultrafiltered (using membranes with molecular weight cut-offs of 100 or 300 kDa). Therefore, whether hydrolyzed or not, most soluble proteins of *Nannochloropsis* species have molecular weights smaller than 100–300 kDa. In fact, Moreira et al. [28] reported that the molecular weights of proteins extracted from *N. oceanica* using several extraction techniques, including HPH and enzymatic hydrolysis, ranged from 10 to 50 kDa.

Despite the similar lipid extraction yields in both biorefineries, the purity of the lipid extract from the EH biorefinery was nearly twice that of the HPH biorefinery. During EH, many proteins and carbohydrates were solubilized by cellulase and released from the cells. In contrast, the limited disruption efficiency of HPH resulted in a greater fraction of these compounds remaining attached to the cells. Polar solvents, such as ethanol, coextract non-lipid polar components, including proteins and carbohydrates, together with the lipid fraction [29,30]. Therefore, some of the water-soluble polar components that were not released during HPH were extracted with ethanol, yielding a relatively low-purity lipid extract. Soto-Sierra et al. [31] reported a higher lipid extraction yield (85%) and purity of lipid extract (85% *w*/*w*) than those obtained in this work for EH biorefinery (31.2 ± 2.3% extraction yield and 72.0 ± 5.3% *w*/*w* extract purity). The authors used a 200 g L^−1^ *Nannochloropsis* sp. slurry, which was not subjected to cell disruption before lipid extraction, as the extraction substrate. This slurry was much more concentrated than the MF retentates used for lipid extraction in this work (28.0 ± 2.9 g L^−1^). The impact of not performing cell disruption before wet lipid extraction, which significantly improves lipid recovery [32], is counterbalanced by the higher ethanol volumetric concentration used in the extraction (90% *v*/*v*) compared to that used in this work (58.2 ± 2.5% *v*/*v*). In addition, Soto-Sierra et al. [31] conducted a two-stage extraction, each lasting 45 min at 60 °C. In contrast, this study performed a single one-hour extraction at room temperature, which may help explain the observed differences.

Despite the coextraction of non-lipid polar compounds in the ethanolic extraction of the HPH biorefinery, the majority of the proteins and carbohydrates of the biomass were not extracted in either water or ethanol and were recovered in the insoluble fraction. In contrast, protein and carbohydrate recovery yields in the insoluble fraction in the EH biorefinery were considerably lower due to the high hydrolytic capacity of cellulase, which promoted the release of water-soluble components from the cells. The EH may also hydrolyze insoluble bioconjugated compounds (e.g., glycoproteins) into more soluble molecules, thereby explaining the higher protein extraction yields of EH biorefinery.

Most mass balances closed to about 90–100%, except for the lipid and carbohydrate balances of the HPH biorefinery due to underestimating the lipid and carbohydrate contents of the insoluble fraction. This indicates that the two-step sulfuric acid hydrolysis and the multi-stage pretreatment performed before carbohydrate and lipid analysis, respectively, were not sufficiently intense to hydrolyze all carbohydrates and expose the lipids to the extraction solvent.

### 3.4. Optimization of Lipid Extraction

The efficiency of wet lipid extraction could be increased by reducing the water-to-ethanol volumetric ratio. Compared with lipid extractions of the EH biorefinery (average ethanol concentration of 58.2 ± 2.5% *v*/*v*), the lipid extraction yield augmented from 31.2 ± 2.3% to 40.0 ± 3.8% (67% *v*/*v* ethanol) and to 57.4 ± 3.1% (75% *v*/*v* ethanol) without significant effects on the purity of the lipid extracts (72.0 ± 5.3% *w*/*w* for 58.2 ± 2.5% *v*/*v* ethanol, 70.8 ± 5.4% *w*/*w* for 67% *v*/*v* ethanol, and 78.9 ± 3.9% *w*/*w* for 75% ethanol). An even larger lipid yield was obtained in a larger-scale extraction performed in a mixing tank (70.1%), without compromising extract purity (80.4% *w*/*w* lipids). This suggests that mass-transfer limitations arose from mixing rather than from solvent lipid saturation or from a short extraction time. Further improvements in lipid extraction could be achieved through multi-step extractions, increased extraction temperature, and higher ethanol volumetric concentrations, as reported by Soto-Sierra et al. [31] for *Nannochloropsis* sp.

Despite the absence of water, the 100% *v*/*v* ethanol extractions performed similarly to the 75% *v*/*v* ethanol extractions, resulting in only a slight increase in the process selectivity. The lipid extraction efficiency (49.4 ± 1.8%) was close to that obtained by Ryckebosch et al. [29] (52%) and Servaes et al. [30] (49%). However, the lipid purities of the extracts (84.7 ± 2.1% *w*/*w*) were significantly higher than those obtained by Ryckebosch et al. [29] (60% *w*/*w*) and Figueiredo et al. [33] (42.4 ± 3.8% *w*/*w*), who used ethanol-to-biomass ratios (60 mL/g DW and 20 mL/g DW, respectively) larger than the 10 mL/g DW ratio used in this work.

### 3.5. Biochemical Composition

#### 3.5.1. Fatty Acid Profile

The fatty acid profile of the lipid extract obtained in this work was similar to that obtained by Figueiredo et al. [33], who also extracted lipids from *Nannochloropsis oceanica* using ethanol as the extraction solvent. The EPA content of the lipid extracts from both biorefineries tested in this work was much higher than the reported range (1–3% *w*/*w*) in the literature [29,33]. Similarly, the fatty acid content of the lipid extracts was considerably higher than the values (20–22% *w*/*w*) reported by Callejón et al. [32]. These authors investigated the impact of water content and the ethanol-to-biomass ratio on the SL extraction yield. These two variables reflect the volumetric ethanol-to-water ratio in each lipid extraction. For example, using 50 mL of ethanol/g DW on a 60 g/L substrate corresponds to an ethanol-to-biomass ratio of 3:1, or 75% *v*/*v*. Callejón et al. [32] performed several wet extractions with ethanol-to-water ratios ranging from 73.7 to 93.5% (*v*/*v*) and demonstrated that higher ratios yield higher SL extraction yields (50.3–98.3%). These observations are consistent with our results, which show that the SL yield increased from 49.6 ± 5.2% to 81.8 ± 3.5% by increasing the ethanol–water ratio from 58.2 ± 2.5% *v*/*v* to 75% *v*/*v* in the EH biorefinery. Interestingly, the SL yield (61.6 ± 3.0%) of the dry extraction (100% *v*/*v* ethanol) was lower than that of the 75% *v*/*v* ethanol extraction, probably because of the low ethanol-to-biomass ratio (10 mL/g DW) used. Callejón et al. [32] obtained considerably higher SL yields from dry extractions than from wet biomass (73.7% *v*/*v* ethanol; 50.3% SL yield), using ethanol–biomass ratios of 20 mL/g DW (92.3% SL yield) and 40 mL/g DW (99.5% SL yield). Cell disruption facilitates solvent access to saponifiable lipids within cells [32]. The higher SL yield of EH biorefinery (49.6 ± 5.2%) compared to the HPH biorefinery (33.2 ± 1.8%) is another indicator of EH’s higher permeabilization efficiency than HPH (despite similar lipid extraction yields).

#### 3.5.2. Amino Acid Profile

In contrast to animal proteins, most plant proteins contain relatively low levels of EAAs and are considered incomplete protein sources [34]. Some exceptions are quinoa, soy, buckwheat, or amaranth [35,36]. All fractions obtained from *N. oceanica* biomass in both biorefineries exhibited a complete EAA profile, with overall EAA content exceeding the WHO/FAO/UNU values proposed for a balanced protein source [19]. Except for tryptophan, all other EAAs were present at higher concentrations in each produced fraction than in the requirement pattern. The fractionation process yielded fractions with absolute EAA contents that were significantly different from those of the original lysates. Insoluble fractions presented the highest percentage of EAAs from total amino acids (46% in both biorefineries), and UF permeates the lowest percentage of EAAs from total amino acids (39% in both biorefineries). Thus, protein-rich fractions, whether water-soluble or insoluble, could be incorporated into human food and animal feed or used as nutritional supplements for humans.

### 3.6. Preliminary Economic Analysis

The extent of dewatering of microalgae culture after harvesting is significant for the economics of the biorefinery. Membrane-based concentration methods consume less energy than cell disruption techniques, such as high-pressure homogenization, especially for biomass with recalcitrant cell walls. Therefore, reducing volume is essential to lower energy consumption. Even for less energy-intensive processes such as enzymatic hydrolysis, heating the culture from room temperature to 50 °C (the optimal temperature for cellulase) and mixing require significantly less energy when the volume is reduced five-fold. Conversely, only a small additional energy input (approximately 4%) is required to further concentrate the culture from 20 to 100 g L^−1^, representing just a 5-fold increase. Most of the volume reduction (a 20-fold decrease) occurred during concentration from approximately 1 g L^−1^ to 20 g L^−1^.

Across all simulated scenarios, disrupting *N. oceanica* cells with EH instead of HPH resulted in lower lipid production costs. It is important to note that the energy required to heat the concentrated microalgae culture before EH was not included in the calculations. Only the power needed for mixing and maintaining a temperature of 50 °C during EH was considered. Nonetheless, enzymatic hydrolysis, often considered costly on an industrial scale due to enzyme costs, proved highly effective in breaking down the *N. oceanica* cell wall, thereby improving downstream processing.

Scaling up the process tenfold yielded only a 26% cost reduction in scenario 5, compared to 37% and 39% reductions in scenarios 3 and 4, respectively. This is because the pilot spray dryer is less energy-efficient than the lab spray dryer. Typically, larger-scale equipment consumes less energy per unit of capacity, but this trend does not apply to these two Pilotech spray dryers. With a 70% extraction yield from the scaled-up lipid extraction process in a mixing tank, an additional 18% reduction in lipid production costs (from 40 to 33 €/kg) could be achieved (scenario 4).

## 4. Materials and Methods

### 4.1. Microalga

*Nannochloropsis oceanica* was supplied by Allmicroalgae-Natural Products (Leiria, Portugal) as a concentrated frozen paste (≈20% *w*/*w* dry weight). This biomass was cultivated in outdoor tubular photobioreactors under autotrophic conditions at the company’s production facility in Pataias, Portugal. After harvesting, the culture was pre-concentrated by membrane microfiltration (0.2 µm membrane pore size) followed by centrifugation (7500× *g*). The concentrated algae suspension was immediately frozen to preserve the biomass’s original properties.

### 4.2. Biorefinery Processes

#### 4.2.1. Cell Disruption

**Figure A1:**
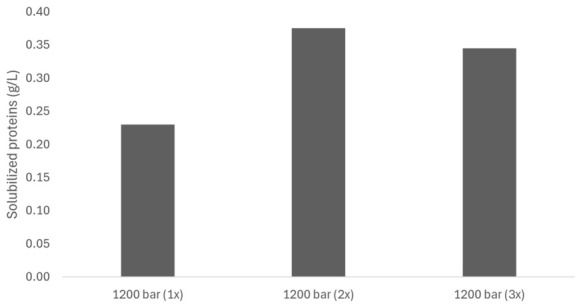
Preliminary assay assessing the influence of the number of high-pressure homogenization cycles at the maximum pressure (1200 bar) on the amount of protein extracted from *N. oceanica* cells (*n* = 1). The soluble protein content after high-pressure homogenization was normalized to the pre-homogenization value.

**Figure A2:**
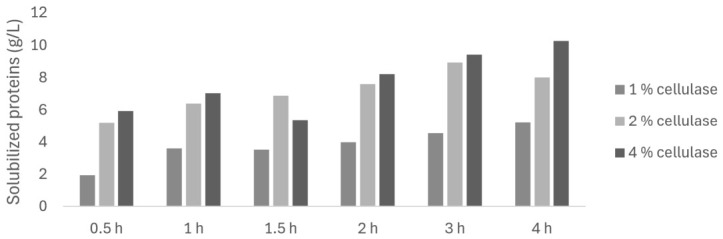
Preliminary assay assessing the effects of enzyme dosage and time on the amount of protein extracted from *N. oceanica* cells during cellulase-mediated enzymatic hydrolysis (*n* = 1). The soluble protein content after enzymatic hydrolysis was normalized to the pre-hydrolysis value. The total mass of cellulase added was subtracted from the total mass of soluble proteins obtained after enzymatic hydrolysis.

*N. oceanica* suspensions with 100 g L^−1^ were prepared from the frozen microalgal paste and processed by both high-pressure homogenization and enzymatic hydrolysis. HPH consisted of two cycles of homogenization at 1200 bar using a Panda Plus 2000 homogenizer (GEA Niro Soavi, Parma, Italy). Cellulyve AN 3500 (Solyve, Caen, France), a commercial cellulase preparation, was used for hydrolysis at 2% (*w*/*w*). The enzyme catalyzes the decomposition of cellulose by breaking β-1,4-glycosidic bonds and has an enzymatic activity (using carboxymethylcellulose as the substrate) of 3500 ± 200 U/g. The cells were incubated at 50 °C and a pH of 4.5 for 3 h. After enzymatic hydrolysis, the hydrolysate was refrigerated at 4 °C to inactivate cellulase activity. A preliminary optimization for both disruption methods was performed (see Figure A1 and Figure A2 in Appendix B).

#### 4.2.2. Membrane Filtration

The integrated membrane filtration process was developed in a previous study by our research group [37]. After cell disruption, the homogenate/hydrolysate was clarified by tangential-flow microfiltration (MF) using a 0.2 µm pore-size PVDF membrane (Synder Filtration, Vacaville, CA, USA). Initially, MF was operated in diafiltration mode by adding two diavolumes of water using a peristaltic pump. At this stage, the volume on the retentate side of the membrane remained constant, as the water flux was equal to the permeate flux. MF was operated in a concentration mode after diafiltration. Following, tangential-flow ultrafiltration (UF) with a 100 kDa molecular weight cut-off (MWCO) PES membrane (Synder Filtration, Vacaville, CA, USA) was performed to concentrate the MF permeate and obtain a protein concentrate.

Both MF and UF were operated at a constant permeate flux of 12 L m^−2^ h^−1^ [18] using Vibro-Lab3500 membrane systems (Sani Membranes, Farum, Denmark) with a 0.35 m^2^ filtration area. The manometric pressure inside the membrane module was monitored during each filtration process and never exceeded 0.2 bar (well below the system’s maximum manometric pressure of 3 bar). It was maintained in the 0.1–0.15 bar range to ensure membrane cartridge expansion and reduce fouling.

Membrane cleaning consisted of an initial hot-water rinse (50 °C), followed by an alkaline detergent cleaning (50 °C, pH 10, 30 min) to remove organic matter attached to the membrane, an acid cleaning (50 °C, pH 2, 15 min) to remove inorganic deposits, and filling with a 20% *v*/*v* ethanol storage solution, as recommended by the supplier.

#### 4.2.3. Lipid Extraction

Lipids were extracted with 96% (*v*/*v*) ethanol. The retentates from MF served as substrates for extraction and were used directly, without drying. The ethanol-to-biomass ratio was 50 mL/g dry weight (g-DW). Each extraction involved 3 g of retentate dry weight. Extractions were carried out for 1 h in 500 mL Schott bottles, with magnetic stirring at 300 rpm.

Lipid extraction was optimized by varying the volumetric ethanol concentration. Retentates from MF were dried and resuspended in water to achieve ethanol concentrations of 50, 67, 75, and 100% (*v*/*v*). The ethanol-to-biomass ratios were 50 mL/g-DW (50%, 67%, and 75% *v*/*v* ethanol) and 10 mL/g-DW (100% *v*/*v* ethanol). Extractions were carried out for 1 h in 50 mL Erlenmeyer flasks, with magnetic stirring at 250 rpm. The lipid extraction (75% *v*/*v*) was scaled up and conducted in a 30 L mixing tank agitated at 300 rpm. A scale-up factor of approximately 13 was employed (40 g-DW of retentate processed). The optimization and scale-up of lipid extraction were conducted exclusively for the EH biorefinery.

All the ethanolic extractions were carried out at room temperature.

### 4.3. Dry Weight Quantification

The dry weight was determined by adding known volumes of the sample to pre-weighed plates and oven-drying them overnight at 100 °C. Plates were then transferred to a desiccator for at least 30 min before weighing on a precision balance (GH-202, A&D Instruments, Abingdon, UK).

### 4.4. Biochemical Analyses

#### 4.4.1. Protein Quantification

The protein content was assessed using multiple methods, including the Lowry assay, total nitrogen analysis, and total amino acid profiling. The Pierce™ Modified Lowry Protein Assay Kit (Thermo Fisher Scientific, Waltham, MA, USA) was employed according to the manufacturer’s instructions to measure proteins in water-soluble fractions. The total nitrogen content of each produced fraction was determined by the Dumas combustion method using an organic elemental analyzer (Vario EL III, Elementer, Langenselbold, Germany), according to the manufacturer’s protocol. Total amino acids were analyzed only for the fractions from the third biological replicate of the biorefinery. Freeze-dried samples were hydrolyzed with 6 N HCl at 121 °C for 72 h. For tryptophan analysis, alkaline hydrolysis using 4 N NaOH at 116 °C for 72 h was performed. After cooling, the extracts were concentrated to dryness using a speed-vacuum system (Concentrator plus, Eppendorf SE, Hamburg, Germany), neutralized, and resuspended in 0.1 N HCl. They were then derivatized following the Waters Ultra AccQ-Tag™ protocol for hydrolysate amino acids and quantified by Ultra Performance Liquid Chromatography equipped with an SCL-40 system controller and an SPD-M40 photodiode array detector (Shimadzu Corporation, Kyoto, Japan). Chromatographic conditions followed the guidelines of the certified Waters AccQ-Tag™ method for hydrolysate amino acids.

K_P_ nitrogen-to-protein conversion factors were calculated using the total protein content (measured as the sum of all amino acid residues) and the total nitrogen content (as defined by Sriperm et al. [38]) for each fraction of one of the biological replicates from the biofineries tested. An average nitrogen-to-protein factor of 5.35 was then used to estimate the protein content of all fractions produced across the three replicates of the biorefinery process and to determine the protein mass balances.

Non-protein nitrogen was determined by subtracting protein nitrogen from total nitrogen. Protein nitrogen was calculated based on the amino acid residue composition and the nitrogen content of each amino acid residue [23].

#### 4.4.2. Carbohydrate Quantification

The fractions containing non-water-soluble compounds, such as homogenates, hydrolysates, microfiltration retentates, and final insoluble fractions, were subjected to a two-step sulfuric acid hydrolysis before soluble carbohydrate quantification, as described by Van Wychen & Laurens [39]. Approximately 10 mg of freeze-dried, well-ground powdered fractions were hydrolyzed in 250 µL of 72% *w*/*w* sulfuric acid at 30 °C for 1 h. Then, the solution was diluted to 4% *w*/*w* sulfuric acid by adding 7 mL of Milli-Q water and autoclaved for 1 h at 121 °C. After the second hydrolysis, the samples were allowed to cool to room temperature, then centrifuged (2000× *g*, 10 min, 4 °C) to remove all solids and obtain clear, soluble extracts. Permeates from MF and UF, and the retentates from UF, were analyzed directly without any pretreatment.

The soluble carbohydrate content was measured using the phenol–sulfuric acid method [40]. A 0.5 mL volume of phenol (5% *v*/*v*) was added to glass tubes containing an equal volume of the previously diluted fractions. Then, 2.5 mL of sulfuric acid (96% *v*/*v*) was added directly onto the liquid surface, avoiding contact with the tube walls, to ensure proper mixing. After 10 min, the samples were vortexed and incubated for an additional 30 min before absorbance was measured at 490 nm. A calibration curve was created using glucose as the standard.

#### 4.4.3. Lipid Quantification

Before lipid extraction, the samples underwent multi-stage pretreatment. Approximately 20 mg of the previously lyophilized fractions were resuspended in 0.8 mL of ultrapure Milli-Q^®^ water and frozen overnight. After defrosting, the suspensions were placed in an ultrasonic cleaner (USC 300 T, VWR International, Radnor, PA, USA) for 5 min and subjected to three cycles (1 min per cycle, with 30 s intervals between cycles to prevent sample overheating) in a mixer mill (MM 400, Retsch GmbH, Haan, Germany) at 25 Hz. Ethanolic extracts did not require any pretreatment. Instead, extracts were transferred to pre-weighed tubes and oven-dried overnight at 100 °C. The tubes were subsequently transferred to a desiccator for at least 30 min and weighed using a precision microbalance (MSA36S-000-DH, Sartorius Lab Instruments GmbH & Co. KG, Göttingen, Germany).

The total lipid content was determined by gravimetric analysis using the Bligh and Dyer method [41] with slight modifications. First, a monophasic ternary solvent system consisting of methanol, chloroform, and ultrapure Milli-Q water (2:1:0.8 volume ratio) was used to extract both polar and non-polar lipids. Then, a biphasic system was created by adjusting the solvent ratio (2:2:1.8) with the addition of water and chloroform. The majority of lipids were in the chloroform (lower) phase, and most of the non-lipid components, such as proteins and carbohydrates, were in the methanol–water (upper) phase. After centrifugation (2500× *g*, 10 min), 0.5 mL of the chloroform fraction was transferred to pre-weighed tubes and placed overnight in a dry bath incubator (DBH 2 S000, IKA-Werke GmbH & Co. KG, Staufen, Germany) at 55 °C to evaporate the solvent.

#### 4.4.4. Fatty Acid Profile

Before fatty acid derivatization and extraction, the samples underwent the same multi-stage pretreatment as described in the previous section. Ethanolic extracts did not require any pretreatment. Instead, the extracts were transferred to pre-weighed tubes and dried overnight under a gentle nitrogen flow. The tubes were subsequently transferred to a desiccator for at least 30 min and weighed using a precision microbalance (MSA36S-000-DH, Sartorius Lab Instruments GmbH & Co. KG, Göttingen, Germany).

Fatty acids were determined after conversion to fatty acid methyl esters (FAMEs) using a modified Lepage and Roy method as described by Pereira et al. [42]. FAMEs were analyzed using a gas chromatography–mass spectrometer equipped with a SCION 456-GC and a SCION TQ MS (SCION Instruments, Livingston, UK). The gas chromatograph was equipped with a 30 m column with an internal diameter of 0.25 mm and a 0.25 μm film thickness (ZB-5MSplus, Phenomenex Inc., Torrance, CA, USA). Helium was the carrier gas. The temperature program was 60 °C (1 min), 30 °C min^−1^ to 120 °C, 4 °C min^−1^ to 250 °C, and 20 °C min^−1^ to 300 °C (4 min). The total running time was 42 min. The injection temperature was 300 °C. Supelco^®^ 37 Component FAME Mix (Supelco, Sigma-Aldrich, Bellefonte, PA, USA) was used as the standard. After identification and quantification, the fatty acid profile was expressed as a relative percentage of the total fatty acid content present in the biomass.

### 4.5. Mass Balances

All fractions were analyzed, and mass balances were calculated on a moisture-free basis to determine the recovery of each biomass component at every step of the cascading process.

### 4.6. Statistical Analysis

Both biorefinery processes, involving cell permeabilization either through high-pressure homogenization or enzymatic hydrolysis, were conducted in triplicate. The figures and tables display the average values along with their standard deviations. The results were statistically compared at the *α* = 0.05 significance level using paired samples. The difference between paired sample values was calculated, and normality was assessed using the Shapiro–Wilk test. When the data met the assumption of normality, a paired *t*-test was used. If the assumption of normality was violated, the Wilcoxon Signed-Rank test was applied. For lipid extraction optimization, the data (independent samples) were analyzed using one-way analysis of variance (ANOVA), followed by Tukey’s multiple-comparison tests. When the assumptions of normality and homogeneity of variance were violated, the Kruskal–Wallis and Welch’s ANOVA tests were performed. All analyses were conducted in R (version 4.2.2) using RStudio IDE (version 2025.09.1-401).

### 4.7. Preliminary Economic Analysis

The energy consumption for each process stage was estimated based on the equipment’s capacity and power requirements (Table 7). The energy required to recover ethanol after lipid extraction was estimated using the specific heat capacities and latent heats of vaporization of ethanol and water. This is a rough, simplified estimate used only for comparison purposes, as accurately quantifying distillation energy consumption requires proper process simulation software. Dewatering by membrane filtration, performed after culture harvesting and before biorefinery processing, was also included in the economic evaluation to integrate the upstream and downstream processing. Several scenarios were developed with different sequences of processes and operational conditions (Table 8). The production costs of lipids (for lab-scale processing of 0.5 kg of *N. oceanica* biomass) were calculated for each scenario based on energy consumption (0.16 €/kWh), enzyme use (10 €/kg), and yields at each process stage in the biorefinery. A ten-fold scale-up factor was then applied to evaluate the effects of scale on the production costs (Table 7).

## 5. Conclusions

Combining enzymatic hydrolysis, a cell-disruption method specifically targeting the *Nannochloropsis* cell wall, with subsequent downstream processes such as membrane filtration and ethanolic extraction proved more effective than a biorefinery based on high-pressure homogenization. The hydrolytic action of cellulase resulted in the solubilization of 48.2 ± 1.5% of the total biomass compared with only 27.3 ± 3.2% using HPH. This, along with the similar total mass recovery yields (12.9 ± 0.8% for HPH biorefinery and 15.1 ± 1.6% for EH) in both ethanolic extractions, led to an insoluble fraction comprising 66.1 ± 2.1% of the initial *N. oceanica* biomass for HPH biorefinery, significantly higher than that obtained in the EH biorefinery (44.9 ± 1.4%). The lipid production cost through the EH biorefinery (101 €/kg) was lower than that of the HPH biorefinery (165 €/kg). It could be further reduced to 40 €/kg by optimizing lipid extraction and a tenfold scale-up. The extracts with higher lipid (72.0 ± 5.3% *w*/*w*) and EPA (28.1 ± 6.9% *w*/*w*) concentrations were obtained after EH.

Overall, this work addressed major bottlenecks in *Nannochloropsis* downstream processing, including cell wall recalcitrance to disruption and the dry-versus-wet processing dilemma. In addition, it provided insights into accurate protein quantification (particularly the underestimation of protein content in water-soluble fractions using the Lowry quantification method) and its relevance to the development of integrated fractionation processes for protein-rich biomasses. Future work should focus on investigating the protein molecular weight profile of soluble fractions to refine protein fractionation and obtain a highly concentrated fraction upon ultrafiltration. Furthermore, research on the stabilization of the final biorefinery fractions (by solvent evaporation) and on product formulation is needed to turn these fractions into viable products.

## Figures and Tables

**Table 1:** Protein quantification of water-soluble fractions after disruption of *Nannochloropsis oceanica* cells, determined using their total amino acid content or by using the modified Lowry method. The values are presented as percentages of the fraction’s dry weight (*n* = 1).

Disruption Process	Fraction	Total AA (% *w*/*w*)	Lowry (% *w*/*w*)
High-pressure homogenization	MF permeate	17.2	5.6
	UF permeate	14.2	3.4
	UF retentate	24.2	16.2
Enzymatic hydrolysis	MF permeate	28.1	25.4
	UF permeate	24.8	22.1
	UF retentate	22.4	29.5

**Table 2:** Nitrogen-to-protein conversion factors for each fraction of the two biorefineries applied to *Nannochloropsis oceanica* biomass (*n* = 1). These values represent the ratio of total amino acid to total nitrogen content in each fraction. The percentage of non-protein nitrogen (NPN) relative to total nitrogen is also shown.

Fraction	High-Pressure Homogenization		Enzymatic Hydrolysis	
	N-Protein Ratio	NPN (%)	N-Protein Ratio	NPN (%)
Lysate	5.45	2.7	5.35	7.6
MF permeate	5.37	10.6	5.43	1.7
MF retentate	5.38	5.7	5.47	16.7
UF permeate	5.28	6.6	5.46	3.6
UF retentate	5.23	10.5	3.92	33.2
Lipid extract	5.22	6.0	5.24	5.7
Insoluble fraction	5.34	10.7	5.35	10.4

**Table 3:** Overall mass balances for both biorefineries, covering total biomass, proteins, lipids, and carbohydrates. The values represent the mass recovery yields for each of the final fractions, calculated from the total biomass, proteins, lipids, and carbohydrates in the lysates. The sum of the mass contribution of each fraction relative to each biomass component is also presented. The values are presented as the average and standard deviation of three biological replicates. * Indicates statistically significant differences (*p* < 0.05) between both permeabilization strategies within a fraction.

% *w*/*w*	Total Biomass		Proteins		Lipids		Carbohydrates	
	HPH	EH	HPH	EH	HPH	EH	HPH	EH
UF permeate	17.7 ± 1.7	37.0 ± 3.0 *	6.1 ± 0.6	28.5 ± 3.9 *	0.7 ± 0.4 ^2^	1.7 ± 1.0 ^2^	3.0 ± 0.6	54.4 ± 2.2 *
UF retentate	5.0 ± 1.5	10.5 ± 1.2 *	3.0 ± 0.7	10.6 ± 3.0	0.2 ± 0.1	0.4 ± 0.2 ^2^	2.6 ± 0.5	13.9 ± 3.4 *
Lipid extract	9.7 ± 0.5	8.0 ± 0.8 *	5.8 ± 2.8	2.0 ± 0.6	16.6 ± 5.1	24.9 ± 5.4	-	8.0 ± 3.2 ^1^
Insoluble fraction	66.1 ± 2.1	44.9 ± 1.4 *	82.2 ± 6.6	55.8 ± 3.1 *	33.8 ± 14.0	52.3 ± 12.9	62.8 ± 13.2	22.8 ± 1.8
Total	98.5 ± 4.2	100.4 ± 3.5	97.1 ± 9.0	97.0 ± 9.1	64.3 ± 1.3	91.5 ± 5.2	82.7 ± 7.7	99.1 ± 7.4

^1^ The mass of carbohydrates in the lipid extracts was calculated as the difference between the mass of carbohydrates in the MF retentate and in the insoluble fraction. ^2^ Average and standard deviation of only two biological replicates.

**Table 4:** Biochemical composition of the *N. oceanica* biomass and the fractions resulting from both biorefineries tested. The protein, lipid, and carbohydrate contents (in % *w*/*w*) are shown. The values are presented as the average and standard deviation of three biological replicates. * Indicates statistically significant differences (*p* < 0.05) between both permeabilization strategies within a fraction.

% *w*/*w*	Proteins		Lipids		Carbohydrates	
	HPH	EH	HPH	EH	HPH	EH
Initial biomass	33.7 ± 2.8		24.4 ± 6.7		19.2 ± 2.3	
UF permeate	12.0 ± 2.9	24.7 ± 1.2 *	0.9 ± 0.6 ^2^	1.0 ± 0.7 ^2^	3.2 ± 0.5	28.1 ± 1.9
UF retentate	21.2 ± 3.4	31.9 ± 4.9 *	1.4 ± 0.8	0.7 ± 0.5 ^2^	10.8 ± 2.7	25.2 ± 4.6 *
Lipid extract	19.6 ± 9.1	8.2 ± 2.2	38.8 ± 6.1	72.0 ± 5.3 *	-	18.9 ± 7.2 ^1^
Insoluble fraction	41.6 ± 1.1	40.0 ± 1.2	14.4 ± 8.2	28.7 ± 2.6	18.5 ± 5.3	9.7 ± 1.4

^1^ The mass of carbohydrates in the lipid extracts was calculated as the difference between the mass of carbohydrates in the MF retentate and in the insoluble fraction. ^2^ Average and standard deviation of only two biological replicates.

**Table 5:** Total saponifiable lipids (SL) and EPA contents (in % *w*/*w*) and ratio between polyunsaturated fatty acids (PUFA) and saturated fatty acids (SFA) of *Nannochloropsis oceanica* biomass and the fractions obtained after separating the water-soluble compounds in both biorefineries (HPH and EH). Two lipid extracts (75% *v*/*v* and 100% *v*/*v*) from the lipid extraction optimization are also represented. Data are presented as the average and standard deviation of three biological replicates.

% *w*/*w*		High-Pressure Homogenization			Enzymatic Hydrolysis				
	Initial Biomass	MF Retentate	Lipid Extract	Insoluble Fraction	MF Retentate	Lipid Extract	Insoluble Fraction	75% *v*/*v* Lipid Extract	100% *v*/*v* Lipid Extract
SL	11.8 ± 3.9	15.3 ± 0.7	40.9 ± 1.7 ^1^	14.4 ± 2.8	25.2 ± 0.5 ^1^	75.5 ± 6.7	26.7 ± 0.4	81.2 ± 5.8	82.5 ± 5.0
EPA	2.7 ± 1.0	4.4 ± 0.2	9.1 ± 0.6 ^1^	3.6 ± 1.0	6.2 ± 0.2 ^1^	28.1 ± 6.9	7.3 ± 0.3	25.7 ± 1.5	27.1 ± 1.5
PUFA/SFA	1.0 ± 0.1	1.4 ± 0.0	1.0 ± 0.0	1.2 ± 0.1	1.0 ± 0.0	2.0 ± 0.4	1.3 ± 0.0	1.5 ± 0.1	1.6 ± 0.0

^1^ Average and standard deviation of only two biological replicates.

**Table 6:** Specific energy use and production costs of the lipid extract from *Nannochloropsis oceanica* across the various biorefinery scenarios described in Table 8, at 0.5 kg and 5 kg production scales.

Scenario	Specific Energy Consumption (kWh/kg Lipids)		Cost of production (€/kg Lipids)	
	0.5 kg	5 kg	0.5 kg	5 kg
1	1243	-	199	-
2	1033	844	165	135
3	614	385	101	64
4	400	239	66	40
5	548	402	90	67

**Table 7:** Power requirements and capacity of the equipment used at the lab-scale biorefinery (0.5 kg of biomass) and for the upstream membrane filtration dewatering process. The equipment needed for a 10-fold scale-up (5 kg of biomass) is also included.

Process	Equipment	Power (kW)	Capacity	Scale
Dewatering	Vibro-I (10 m^2^) (Sani Membranes, Farum, Denmark)	1.98	20 L m^−2^ h^−1^	0.5 kg
	Vibro-I (100 m^2^) (Sani Membranes, Farum, Denmark)	12.00		5 kg
High-pressure homogenization	Panda Plus 2000 (Niro-Soavi, Düsseldorf, Germany)	1.85	9 L h^−1^	0.5 kg
	Panther 3006 (Niro-Soavi, Düsseldorf, Germany)	5.50	120 L h^−1^	5 kg
Hydrolysis tank	-	2.25	30 L	0.5 kg
		5.25	100 L	5 kg
Microfiltration	Vibro-Lab3500 (Sani Membranes, Farum, Denmark)	0.14	12 L m^−2^ h^−1^	0.5 kg
	Vibro-I (Sani Membranes, Farum, Denmark)	1.98		5 kg
Extraction tank	-	0.25	30 L	0.5 kg
		P/V constant ^1^	>100 L	5 kg
Spray-drying	Lab SD YC-018 (PILOTECH, Shanghai, China)	5.5	3.5 L h^−1^	0.5 kg
	Pilot SD YC-029 (PILOTECH, Shanghai, China)	20	10 L h^−1^	5 kg

^1^ For volumes higher than 100 L, the mixing energy consumption was estimated by maintaining the specific power input (P/V) constant.

**Table 8:** Different biorefinery scenarios for extracting a lipid-rich product from *Nannochloropsis oceanica*. Two different concentration factors were used in membrane filtration processes (dewatering and microfiltration). Cell permeabilization was done either by high-pressure homogenization or enzymatic hydrolysis. Various ethanol volumetric concentrations were applied in the lipid extraction process. The processes occur in the sequence shown for each scenario (from left to right).

Scenario	Dewatering ^1^	HPH	EH	MF ^2^	Spray-Drying	Lipid Extraction	Distillation
1	20 g/L	x	-	40 g/L	-	67% *v*/*v* ethanol	
2	100 g/L	x	-	40 g/L	-	67% *v*/*v* ethanol	
3	100 g/L	-	x	40 g/L	-	67% *v*/*v* ethanol	
4	100 g/L	-	x	60 g/L	-	75% *v*/*v* ethanol	
5	100 g/L	-	x	60 g/L	x	100% *v*/*v* ethanol	

^1^ Final concentration of the microalgae suspension after biomass harvesting and dewatering. ^2^ Concentration of the retentate at the end of the microfiltration.

## Data Availability

The original contributions presented in this study are included in the article. Further inquiries can be directed to the corresponding author.

## Abbreviations

The following abbreviations are used in this manuscript:

WHO	World Health Organization
FAO	Food and Agriculture Organization
UNU	United Nations University
PUFA	Polyunsaturated Fatty Acid
EPA	Eicosapentaenoic Acid
HPH	High-Pressure Homogenization
EH	Enzymatic Hydrolysis
MF	Microfiltration
UF	Ultrafiltration
MWCO	Molecular Weight Cut-Off
DW	Dry Weight
FAME	Fatty Acid Methyl Ester
AA	Amino Acid
NPN	Non-Protein Nitrogen
SL	Saponifiable Lipid
SFA	Saturated Fatty Acid
EAA	Essential Amino Acid
MUFA	Monounsaturated Fatty Acid

## Appendix A

**Table A1:** Fatty acid profile of *Nannochloropsis oceanica* biomass and the fractions obtained after separating the water-soluble compounds in both biorefineries (HPH and EH). The values are shown as the percentage of each fatty acid relative to the total fatty acids in the fraction. Data are presented as the average and standard deviation of three biological replicates.

Fatty Acid	Initial Biomass	High-Pressure Homogenization			Enzymatic Hydrolysis		
		MF Retentate	Lipid Extract	Insoluble Fraction	MF Retentate	Lipid Extract	Insoluble Fraction
C12:0	0.5 ± 0.1	0.7 ± 0.1	0.8 ± 0.1	0.5 ± 0.1	0.8 ± 0.0	0.7 ± 0.1	0.5 ± 0.0
C14:0	6.0 ± 0.4	5.1 ± 0.2	5.7 ± 0.0	4.5 ± 0.0	5.6 ± 0.0	5.1 ± 0.1	4.6 ± 0.1
C15:0	0.4 ± 0.0	0.3 ± 0.0	0.4 ± 0.0	0.3 ± 0.0	0.4 ± 0.0	0.3 ± 0.0	0.3 ± 0.0
C16:1	31.3 ± 0.9	30.5 ± 0.6	41.3 ± 0.8	32.2 ± 0.6	32.2 ± 0.6	32.1 ± 6.0	31.9 ± 0.3
C16:0	22.7 ± 1.8	18.8 ± 0.3	18.2 ± 0.1	20.2 ± 0.5	22.6 ± 0.1	14.5 ± 0.5	20.0 ± 0.3
C17:1	0.2 ± 0.0	0.2 ± 0.0	0.3 ± 0.0	0.3 ± 0.0	0.3 ± 0.0	0.2 ± 0.1	0.3 ± 0.0
C17:0	0.3 ± 0.0	0.3 ± 0.0	0.2 ± 0.0	0.3 ± 0.0	0.4 ± 0.0	0.1 ± 0.0	0.3 ± 0.0
C18:3 (n-6)	0.4 ± 0.0	0.4 ± 0.0	0.3 ± 0.0	0.4 ± 0.0	0.4 ± 0.0	0.4 ± 0.1	0.4 ± 0.0
C18:2c (n-6)	2.6 ± 0.2	2.6 ± 0.1	1.7 ± 0.0	2.6 ± 0.2	2.0 ± 0.0	2.0 ± 0.1	2.2 ± 0.0
C18:1c	6.4 ± 0.4	6.0 ± 0.5	5.2 ± 0.1	6.8 ± 0.7	5.6 ± 0.2	3.9 ± 0.5	5.8 ± 0.1
C18:1t	0.5 ± 0.1	0.5 ± 0.0	0.5 ± 0.0	0.6 ± 0.1	0.5 ± 0.0	0.4 ± 0.1	0.6 ± 0.1
C18:0	1.1 ± 0.1	1.1 ± 0.2	0.6 ± 0.1	1.4 ± 0.5	1.1 ± 0.1	0.4 ± 0.1	1.0 ± 0.1
C20:4 (n-6)	4.0 ± 0.4	3.9 ± 0.1	2.2 ± 0.0	4.2 ± 0.2	3.1 ± 0.1	2.8 ± 0.5	3.7 ± 0.1
C20:5 (n-3)	22.8 ± 2.0	28.7 ± 0.7	22.3 ± 0.5	24.4 ± 2.1	24.5 ± 0.3	36.8 ± 6.7	27.5 ± 0.7
C20:3 (n-6)	0.4 ± 0.0	0.5 ± 0.0	0.3 ± 0.0	0.8 ± 0.1	0.4 ± 0.0	0.3 ± 0.0	0.5 ± 0.0
C24:1	0.4 ± 0.0	0.3 ± 0.0	n.d.	0.6 ± 0.1	0.4 ± 0.0	n.d.	0.4 ± 0.0
Total SFA	31.1 ± 2.3	26.3 ± 0.3	26.0 ± 0.4	27.2 ± 1.0	30.9 ± 0.0	21.2 ± 0.8	26.7 ± 0.3
Total MUFA	38.8 ± 0.6	37.6 ± 0.5	47.2 ± 1.0	40.4 ± 0.7	38.8 ± 0.6	36.6 ± 6.6	39.1 ± 0.4
Total PUFA	30.1 ± 1.9	36.1 ± 0.8	26.8 ± 0.6	32.3 ± 1.6	30.2 ± 0.6	42.3 ± 7.4	34.3 ± 0.6

**Table A2:** Amino acid profile of the lysates and final fractions produced in the third replicate of both biorefineries. The values are presented in milligrams of amino acids per gram of total dry weight of each fraction. Essential amino acids are given in bold.

Amino Acid (mg/g DW)	High-Pressure Homogenization					Enzymatic Hydrolysis				
	Lysate	UF Retentate	UF Permeate	Lipid Extract	Insoluble Fraction	Lysate	UF Retentate	UF Permeate	Lipid Extract	Insoluble Fraction
**Arg**	86.4	48.2	31.8	15.3	67.6	68.3	44.5	55.0	24.5	66.9
**His**	12.6	5.8	3.8	2.7	12.1	10.8	5.0	6.7	4.2	12.0
**Lys**	30.1	17.2	10.4	7.4	31.7	21.6	15.8	18.5	12.3	31.4
**Thr**	12.3	13.4	5.4	1.4	16.7	12.8	12.5	9.0	2.3	16.5
**Ile**	16.4	13.1	5.8	2.7	20.7	14.0	12.3	10.0	4.4	20.5
**Leu**	31.0	19.9	9.4	4.7	35.2	31.2	18.5	16.4	7.4	34.8
**Val**	21.4	14.2	8.5	4.1	29.5	20.1	13.2	15.1	6.7	29.1
**Met**	6.6	5.6	3.8	2.7	12.8	8.0	5.2	6.9	4.2	12.7
**Phe**	24.7	15.7	7.9	3.6	31.2	22.7	14.7	13.9	5.6	30.8
**Cys**	0.5	0.1	0.1	0.4	2.2	0.9	0.1	0.2	0.7	2.2
**Tyr**	13.5	10.1	6.7	3.8	22.9	14.8	9.5	11.9	6.1	22.6
**Asx**	27.1	15.1	8.2	3.1	20.5	26.3	14.0	14.5	4.9	20.3
**Glx**	35.6	22.4	12.9	4.6	34.3	33.6	20.8	22.1	7.4	33.9
**Ala**	19.9	8.1	6.8	3.2	21.2	19.9	7.6	11.8	5.0	20.9
**Gly**	16.2	10.0	5.6	2.2	21.6	15.4	9.2	9.9	3.4	21.4
**Pro**	13.2	15.6	8.6	2.1	28.1	17.4	14.4	15.4	3.3	27.8
**Ser**	9.5	6.1	5.6	2.3	10.4	11.8	5.9	10.1	3.6	10.3
**Trp**	1.7	1.0	0.5	0.3	2.1	1.5	1.0	1.0	0.6	1.9
**Essential AA**	156.8	105.9	55.5	29.7	192.0	142.6	98.0	97.4	47.8	189.6
**Total AA**	378.7	241.6	141.9	66.7	420.8	351.0	224.1	248.3	106.8	415.9
